# Literary Note

**Published:** 1900-12

**Authors:** 


### LITERARY NOTE.

THE well known firm of Reed & Carnrick has again placed in physicians hands a brochure, superior to any heretofore issued, on the different states and conditions of the blood in health and disease, embellished with many very fine colored illustrations. The great interest taken in blood examinations at the present time, the crystalisation of our knowledge into concrete facts, and their adoption to clinical medicine makes this brochure an extremely welcome one to many physicians, who do not care for the larger text-books on this subject.

The plates, illustrating the condition of the reds and whites in chlorosis, pernicious anemia, leukemia and the parasitic blood diseases are well executed and true to science. The reproduction of the triple stain is well rendered and the plates might well adorn some first class work on hematology.

Those physicians who have not yet received a copy of this brochure may obtain one by addressing the publishers.

